# Dural Injury During Spinal Surgery and Postoperative Cerebrospinal Fluid Leakage: A Single-Center Experience

**DOI:** 10.7759/cureus.71878

**Published:** 2024-10-19

**Authors:** Hayato Kinoshita, Michio Hongo, Takashi Kobayashi, Yuji Kasukawa, Kazuma Kikuchi, Daisuke Kudo, Ryota Kimura, Yuichi Ono, Fumihito Kasama, Naohisa Miyakoshi

**Affiliations:** 1 Orthopaedics, Honjo Daiichi Hospital, Akita, JPN; 2 Physical Therapy, Akita University Graduate School of Medicine, Akita, JPN; 3 Orthopaedic Surgery, Akita Kousei Medical Center, Akita, JPN; 4 Orthopaedic Surgery, Akita University Graduate School of Medicine, Akita, JPN; 5 Orthopaedic Surgery, Yuri Kumiai General Hospital, Yurihonjo, JPN

**Keywords:** cerebrospinal fluid leakage, dural injury, postoperative complications, retrospective study, spinal surgery

## Abstract

Background: The incidence of dural injury during spinal surgery and postoperative cerebrospinal fluid leakage varies between studies. We examined these rates in our institution.

Methods: Among 4014 patients who underwent a spinal operation between January 2012 and February 2020, 176 experienced an intraoperative dural injury (176 of 4014 cases, 4.3%). Among these, 22 (22 of 176 cases, 12.5%) developed postoperative cerebrospinal fluid leakage.

Results: The cause of dural injury was identified in 74 of 176 patients (42%). The rates of dural injury associated with thoracic, cervical, and lumbar operations were 7.8% (25 of 321 cases), 3.2% (19 of 602 cases), and 4.3% (132 of 3091 cases), respectively. Corresponding rates of cerebrospinal fluid leakage were 28% (seven of 25 cases), 5.3% (one of 19 cases), and 11% (14 of 132 cases), respectively. Most patients who experienced cerebrospinal fluid leakage recovered with bed rest; however, cerebral hemorrhage occurred in two patients.

Conclusions: Although it was difficult to identify the cause of dural injury in more than half of patients, suturing the dura and using polyglycolic acid mesh with fibrin glue was effective. No patients required reoperation, even those who developed cerebrospinal fluid leakage.

## Introduction

The reported overall incidence of accidental dural injury during spinal surgery ranges from 1% to 16% and they believe that spinal tumor surgery patients will have a higher incidence of cerebrospinal fluid (CSF) leakage than these [1]. In a multicenter study of 929 patients who underwent posterior spinal fusion, the prevalence of dural injury was 1.9%; however, the rate ranged among centers from 0.6% to 8.5% [2]. The dural injury rate varies widely depending on surgical technique and the specific disease being addressed. CSF leakage is a major complication associated with intraoperative dural injury. Even when the injury is recognized and repaired during the index procedure, CSF leakage requires further treatment in 5% to 10% of cases [3,4]. CSF leakage after dural injury may cause headache, nausea, impaired wound healing, and cerebral hemorrhage. The associated wound healing problems may predispose to infection. These can all prolong hospital stay and increase the economic cost of patient care. Preventing dural injury and establishment of more effective repair and treatment would improve patient care and reduce costs. Numerous studies have examined intraoperative dural injury and postoperative CSF leakage in terms of risk factors and postoperative treatment [5,6]. In this study, we report our experience based on data accumulated over an eight-year period.

## Materials and methods

**Figure 1:**
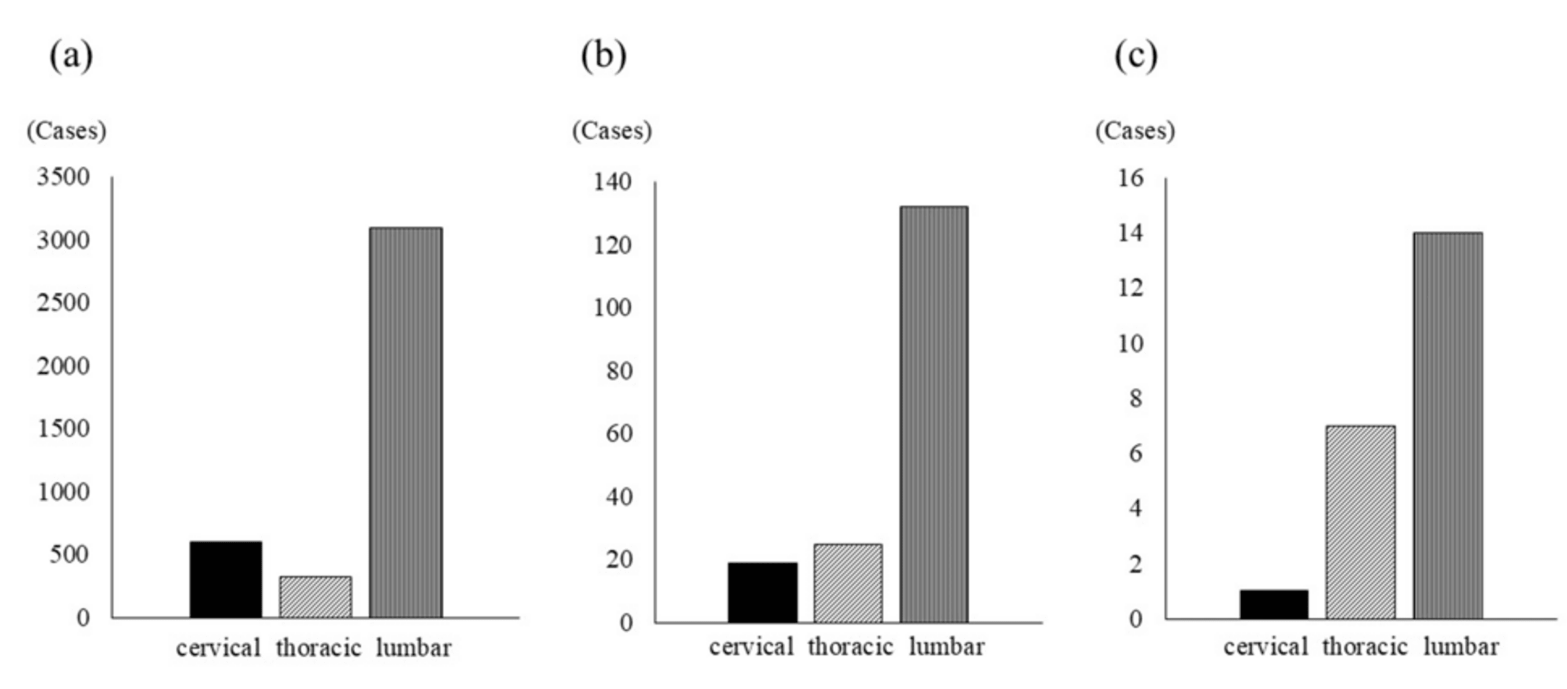
Spinal level (a) It represents the spinal level of all operated patients (n=4014). (b) It represents the spinal level of the patient in whom dural injury has occurred (n=176). (c) It represents the spinal level of the patient in whom CSF leakage occurred (n=22). CSF, cerebrospinal leakage

We retrospectively reviewed the clinical records of 4,014 patients who underwent spinal surgery from January 2012 to February 2020 in our institution. Region of surgery was cervical in 602 patients, thoracic in 321, and lumbar in 3,091 (Figure 1). The 176 patients in this cohort who experienced intraoperative dural injury (4.3%) were analyzed. Dural incision for spinal cord tumor resection was considered a dural injury. Patients with traumatic spine injuries and pre-existing dural tears were excluded to focus on elective spinal surgeries. All intraoperative dural injuries were repaired using primary suturing, polyglycolic acid (PGA) mesh, and fibrin glue. CSF leakage was defined as a confirmed leak of clear fluid from the postoperative wound or by magnetic resonance imaging when swelling around the wound was suspected. All patients provided informed consent in accordance with the Declaration of Helsinki. Institutional ethics committee approval from Akita Kousei Medical Center was obtained (IRB's letter number: 242).

The following data were recorded: type of spinal disease, region of spinal surgery (cervical, thoracic, or lumbar), cause of dural injury, length of hospital stay, postoperative CSF leakage-related complications (headache, infection, cerebral hemorrhage), and treatment of CSF leakage (bed rest alone, bed rest and antibiotics, bed rest and cerebrospinal fluid diversion). The duration of bed rest was until the effusion from the wound stopped. The type of antibiotic used was a cephem antibiotic, assuming infection with bacteria that occurs naturally on the skin. Dressings used on the wound were protected with gauze to absorb moisture and were changed daily until exudate from the wound stopped. Although nausea and vomiting are associated with CSF leakage, they were not examined because these symptoms are common and may also be caused by postoperative narcotic analgesic administration.

Statistical analyses were performed using the open-source Statistical Package for the Biosciences software [7]. Age and length of hospital stay were compared using the unpaired t-test. All other comparisons were performed using the chi-square test. P <0.05 was considered significant.

## Results

Among the 176 patients who experienced intraoperative dural injury, 79 were women and 97 were men. Mean age was 66 ± 13 years (Table 1).

**Table 1:** Patient background CSF, cerebrospinal fluid The data for Age and Hospital stays are expressed as Mean±SD.

	Dural injury (n=176)	Postoperative CSF leakage (n=22)	No postoperative CSF leakage (n=154)	p value
Age (year old)	66±13	65±9	66±13	0.54
Hospital stays (days)	28±26	30±13	28±27	0.60
Sex (Male:Female)	97:79	15:7	82:72	0.42 (χ^2^=1.736)

Postoperative CSF leakage occurred in 22 of 176 patients (12.5%). The indication for surgery was degenerative disease and tumor in a large proportion of cases. Indication for surgery did not significantly differ between the CSF leakage and no CSF leakage groups (Table 2).

**Table 2:** Basic Spinal Diseases CSF, cerebrospinal fluid *, ***p <0.0001 vs. other diseases, chi-square test **p = 0.009 vs. other diseases, chi-square test

	degenerative disease	tumor	trauma	postoperative complication	malformation	infection	p value
Dural injury (n=176)	129 (73%) *	30 (17%)	6 (3%)	5 (3%)	4 (2%)	2 (1%)	<0.0001 (χ^2^=25.153)
Postoperative CSF leakage (n=22)	19 (86%) **	2 (9%)	0	1 (5%)	0	0	0.009 (χ2=6.769)
No postoperative CSF leakage (n=154)	110 (71%) ***	28 (18%)	6 (4%)	4 (3%)	4 (3%)	2 (1%)	<0.0001 (χ2=18.385)

Most surgeries were lumbar, followed by cervical and thoracic. The rates of dural injury (p = 0.004) and CSF leakage (p = 0.03) were significantly higher in thoracic operations than in operations performed in other spinal regions (Figure 1, Table 3).

**Table 3:** Rates of dural injury and cerebrospinal fluid leakage according to region of spinal surgery The thoracic spine showed a tendency to have a higher percentage of dural injury and CSF leakage than the cervical or lumbar spine. CSF, cerebrospinal fluid *p = 0.004 vs. cervical and lumbar, chi-square test **p = 0.03 vs. cervical and lumbar, chi-square test

	cervical	thoracic	lumbar	p value
the ratio of dural injury to each surgery (n=176/4014)	3.2% (19/602)	7.8%* (25/321)	4.3% (132/3091)	0.004 (χ^2^=11.133)
the ratio of CSF leakage in cases with dural injury (n=22/176)	5.3% (1/19)	28%** (7/25)	11% (14/132)	0.03 (χ^2^=6.834)

**Figure 2:**
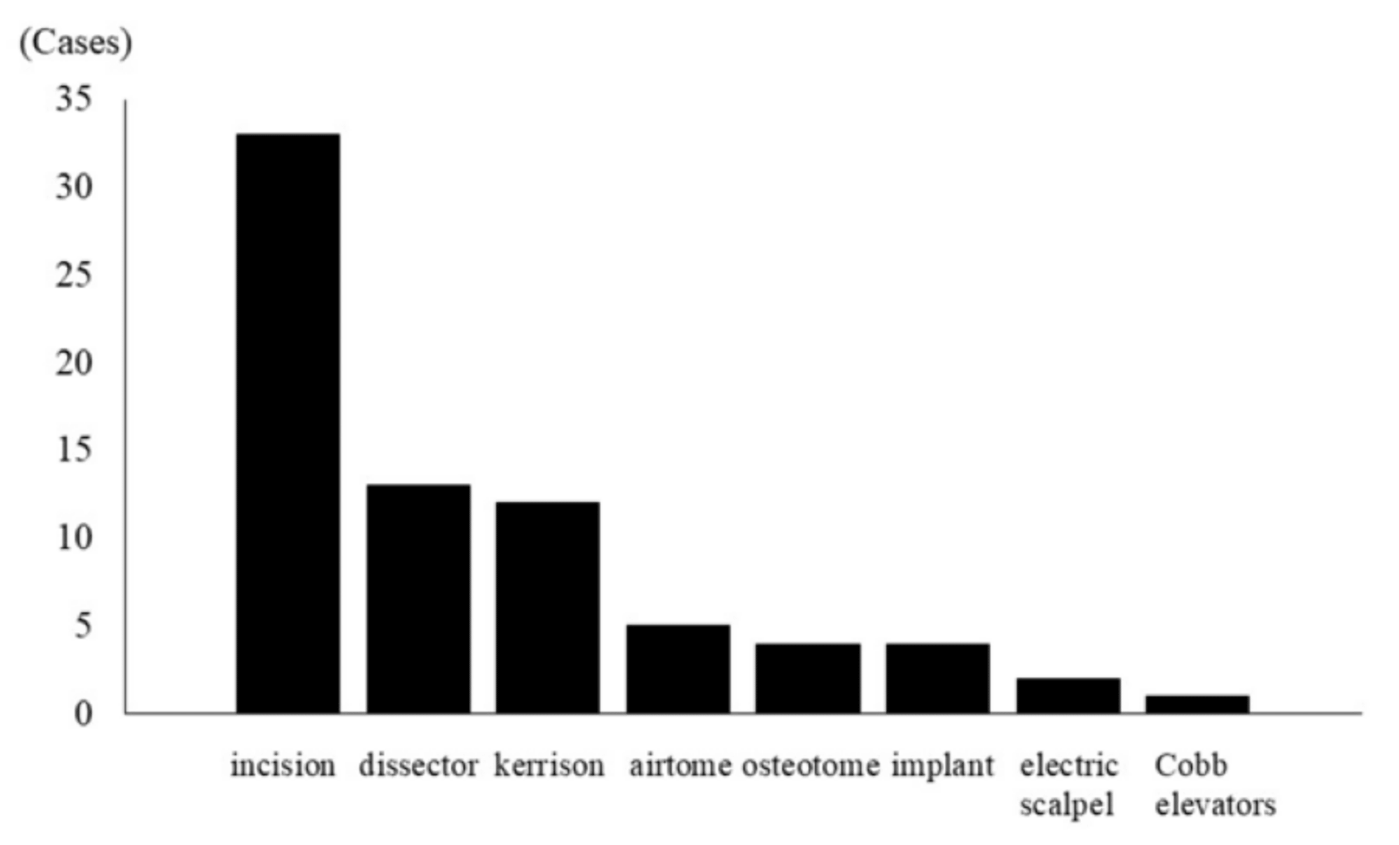
The causes of dural injury Bar chart of the causes of dural injury (n = 74).

The cause of dural injury was identified in 74 of 176 cases (42%). The most common causes, excluding dural incision in 33 of 74 patients who underwent surgery for a spinal cord tumor (44.6%), were a dissecting instrument (13 of 74 patients, 17.6%) and Kerrison rongeur (12 of 74 patients, 16.2%) (Figure 2).

Mean length of hospital stay overall was 28 ± 26 days. Length of stay did not significantly differ between the CSF leakage (30 ± 13 days) and no leakage groups (28 ± 27 days) (Table 1).

The overall incidence of complications in the CSF leakage group was 73% (16 of 22 patients), including headache (eight of 22 patients, 36%), infection (six of 22 patients, 27%), and cerebellar hemorrhage (two of 22 patients, 9%). Treatment of CSF leakage was as follows: bed rest alone in 14 of 22 patients (64%), antibiotics to prevent infection in five of 22 (22%), and cerebrospinal fluid diversion in three of 22 (14%). None of the patients, including those who developed CSF leakage, required reoperation.

## Discussion

**Figure 3:**
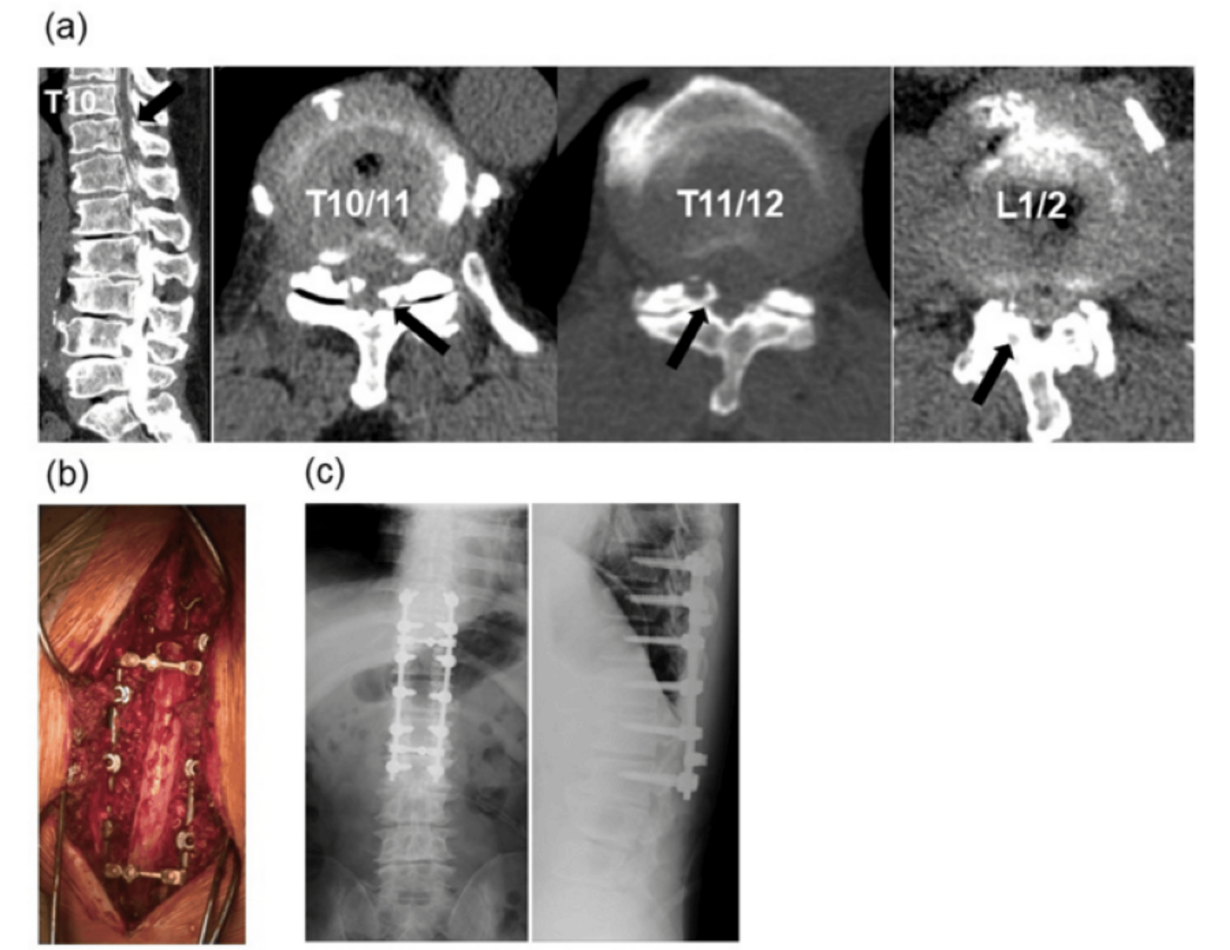
Case presentation of OLF (a) It presents preoperative OLF in the thoracolumbar level (T10/11, T11/12, and L1/2). Black arrows point to OLF. (b) It presents intraoperative image. In this case, no dural injury occurred. (c) It presents postoperative X-ray. We conducted laminectomy from T10 to L2 and a posterior fusion from T9 to L2 in this case. OLF, the ossification of the ligamentum flavum

In our study, most dural injuries occurred in patients undergoing surgery for degenerative disease, mainly because most spinal surgery is performed for this indication. In a previous study, among operations performed for degenerative disease, dural injuries were more frequent in lumbar operations than cervical and thoracic ones [8]. In contrast, we found that the frequency of dural injury was higher in thoracic operations (7.8%) than cervical (3.2%) and lumbar (4.3%) operations; moreover, the incidence of postoperative CSF leakage was higher after thoracic dural injury (28%) than after cervical (5.3%) and lumbar (11%) injuries. The reason for the higher incidence of dural injury in the thoracic spine may be ossification of the posterior longitudinal ligament and the ligamentum flavum, which are relatively common in Asian populations [9,10]. All subjects in our study were Japanese and ossification of the posterior longitudinal ligament was present over the cervicothoracic junction in five patients; ossification of the ligamentum flavum was present in the thoracic spine in seven. When ossified ligaments are resected, large defects can occur in the dura mater, which increases the risk of CSF leakage, even when the dura mater is repaired (Figure 3). Another possible cause is that spinal tumors tend to occur in the thoracic spine. Bezu et al. reported that 27 of 47 patients (57.4%) with spinal tumors had thoracic spine involvement [11]. Spinal tumors are also more likely to be conducted dural resection at tumor resection, and dural injury and CSF leakage may be more likely to occur at this level than at other spinal levels. These factors may have contributed to our results.

Khan et al. reported that among patients undergoing surgery for degenerative lumbar disease, the incidence of dural injury was almost twice as high with revision surgery than with first-time operations (15.9% vs 7.6%) [12]. Baker et al. reported that the strongest risk factor for unintended dural injury in both univariate and multivariate analysis was revision surgery [8]. We did not examine the effect of revision surgery on incidence of dural injury or postoperative CSF leakage and need to do so in the future.

Several previous studies have reported that the main instruments producing intraoperative dural injury in surgeries for degenerative spinal disease are the Kerrison rongeur [13,14]. In our study, the main instruments were the dissector and Kerrison. However, the cause of dural injury could be identified in only 42% of our patients, suggesting that the cause was difficult to identify. Other reported risk factors for dural injury include advanced age, prolonged operation time, and presence of a synovial cyst [6,14].

To prevent dural injury and CSF leakage, Barber et al. recommend that spine surgeons follow basic principles, such as maintaining an adequate field of view, dissecting tissue planes thoroughly before tissue removal, and approaching pathology from areas of normal anatomy when adhesions are present [1]. In our study, each surgeon differed in their approach to the operation and it is not clear whether all of them followed these principles. However, all were skilled and experienced surgeons and we believe they did.

Watertight closure using Gore-Tex suture is effective owing to the absence of a disparity between the diameters of the suture needle and thread [1]. Dafford et al. concluded that 6-0 Prolene was associated with a significantly lower incidence of spinal fluid leakage than 5-0 Surgilon. Furthermore, they noted no difference in results between interrupted and continuous locked sutures [15]. In a previous study, we compared suturing with fibrin glue (20 patients) and suturing with fibrin glue and use of polyglycolic acid mesh (10 patients) as methods of dural repair. CSF leakage occurred in five patients who underwent suturing with fibrin glue; in contrast, no CSF leakage occurred in those who underwent repair using mesh [16]. We mainly use 6-0 Prolene for dural suturing and use both polyglycolic acid mesh and fibrin glue as standard technique.

Postoperative patient positioning after intraoperative dural injury should be upright after cervical operations and flat after lumbar [1]. Although we could not find a clear recommendation for positioning after thoracic dural injury in the literature, we maintain the patient in the upright position after surgery in the upper thoracic spine; after lower thoracic surgery, we place the patient flat. However, after dural injury during lower thoracic spine surgery, we might attempt an upright position if the amount of CSF leakage is high in the flat position.

Although a few patients in our study received antibiotics to prevent infection, antibiotics do not affect the degree of CSF leakage. However, Ulrich et al. noted that the incidence of postoperative wound infection was higher in the durotomy group than in the no durotomy group (6.7% vs. 1.3%) [17]. Prophylactic administration of antibiotics after dural injury does not appear to be unreasonable.

This study has several limitations. First, most cases of CSF leakage were evaluated visually. Only a few underwent magnetic resonance imaging to determine treatment. Second, our study was retrospective in design, so various biases were introduced unlike a prospective study, and had no control group. Third, our results may not be generally applicable, as all our patients were Japanese.

## Conclusions

Dural injury and CSF leakage were more likely with thoracic spine surgery than with surgery in the cervical and lumbar regions. The high incidence of ossification of the posterior longitudinal ligament and the ligamentum flavum in thoracic spine in the Asian population may have resulted in greater surgical dural defects, which may have contributed to the high incidence of postoperative CSF leakage. Adherence to basic dural repair procedures such as primary suturing and use of polyglycolic acid mesh and fibrin glue may prevent CSF leakage which requires reoperation.
